# Dexamethasone and Long-Term Outcome of Tuberculous Meningitis in Vietnamese Adults and Adolescents

**DOI:** 10.1371/journal.pone.0027821

**Published:** 2011-12-08

**Authors:** M. Estée Török, Nguyen Duc Bang, Tran Thi Hong Chau, Nguyen Thi Bich Yen, Guy E. Thwaites, Hoang Thi Quy, Nguyen Huy Dung, Tran Tinh Hien, Nguyen Tran Chinh, Hoang Thi Thanh Hoang, Marcel Wolbers, Jeremy J. Farrar

**Affiliations:** 1 Department of Medicine, University of Cambridge, Cambridge, United Kingdom; 2 Pham Ngoc Thach Hospital, Ho Chi Minh City, Vietnam; 3 Hospital for Tropical Diseases, Ho Chi Minh City, Vietnam; 4 Centre for Clinical Infection and Diagnostics Research, St. Thomas's Hospital, Kings College London, London, United Kingdom; 5 Wellcome Trust Major Overseas Programme, Oxford University Clinical Research Unit, Ho Chi Minh City, Vietnam; 6 Centre for Tropical Medicine, University of Oxford, Oxford, United Kingdom; University of Sassari, Italy

## Abstract

**Background:**

Dexamethasone has been shown to reduce mortality in patients with tuberculous meningitis but the long-term outcome of the disease is unknown.

**Methods:**

Vietnamese adults and adolescents with tuberculous meningitis recruited to a randomised, double-blind, placebo-controlled trial of adjunctive dexamethasone were followed-up at five years, to determine the effect of dexamethasone on long-term survival and neurological disability.

**Results:**

545 patients were randomised to receive either dexamethasone (274 patients) or placebo (271 patients). 50 patients (9.2%) were lost to follow-up at five years. In all patients two-year survival, probabilities tended to be higher in the dexamethasone arm (0.63 versus 0.55; p = 0.07) but five-year survival rates were similar (0.54 versus 0.51, p = 0.51) in both groups. In patients with grade 1 TBM, but not with grade 2 or grade 3 TBM, the benefit of dexamethasone treatment tended to persist over time (five-year survival probabilities 0.69 versus 0.55, p = 0.07) but there was no conclusive evidence of treatment effect heterogeneity by TBM grade (p = 0.36). The dexamethasone group had a similar proportion of severely disabled patients among survivors at five years as the placebo group (17/128, 13.2% vs. 17/116, 14.7%) and there was no significant association between dexamethasone treatment and disability status at five years (p = 0.32).

**Conclusions:**

Adjunctive dexamethasone appears to improve the probability of survival in patients with TBM, until at least two years of follow-up. We could not demonstrate a five-year survival benefit of dexamethasone treatment which may be confined to patients with grade 1 TBM.

**Trial Registration:**

ClinicalTrials.gov NCT01317654 NCT01317654&quest;term&hairsp;&equals;&hairsp;tuberculous&plus;meningitis&amp;rank&hairsp;&equals;&hairsp;3

## Introduction

Tuberculous meningitis (TBM) is the most severe form of *M. tuberculosis* infection and kills or disables more than half of those affected [1]. The use of adjunctive corticosteroids to attenuate the inflammatory response in TBM may improve outcome by reducing the likelihood or severity of neurological complications. Over the past sixty years, a number of clinical studies have investigated this hypothesis using adjunctive corticosteroids in the treatment of TBM [2], [3], [4], [5], [6], [7], [8]. A decade ago a meta-analysis of all randomised controlled trials suggested that adjunctive corticosteroids reduced the risk of death in children but not in adults [9].

We have previously conducted a randomised, double-blind, placebo-controlled trial of adjunctive dexamethasone in 545 Vietnamese adolescents and adults with TBM [1]. Treatment with dexamethasone was associated with a reduction in mortality [relative risk (RR) 0.69, 95% confidence interval (C.I.) 0.52 – 0.92, p = 0.01), but not in neurological disability, compared with placebo. Significantly fewer adverse events occurred in the dexamethasone group than in the placebo group. Although the number of HIV-infected patients included in the study was too small to confidently determine a treatment effect, the use of dexamethasone appeared to be safe in that group of patients.

A more recent meta-analysis of 1140 participants recruited to seven trials has found that corticosteroids reduced the risk of death (RR 0.78, 95% CI 0.67–0.91)[10]. Data on disabling residual neurological deficit from 720 participants in three trials showed that corticosteroids reduce the risk of death or disabling residual neurological deficit (RR 0.82, 95% CI 0.70 to 0.97). The authors concluded that corticosteroids should be routinely used in HIV-uninfected patients with TBM to reduce death and disabling residual neurological deficit amongst survivors. Consequently, adjunctive corticosteroids have become recommended as routine therapy for all patients with TBM [10], [11], [12].

There are limited data on the long-term outcome of patients with TBM [13], [14]. The mechanisms by which corticosteroids reduce mortality remain obscure [15]. It is unknown how long the mortality benefit persists for, or whether the lack of effect on disability will have long-term consequences. Therefore we conducted a follow-up study to determine whether adjunctive dexamethasone was associated with reduced mortality or neurological disability five years after entry into the original trial.

## Methods

### Study design, setting and participants

The original study was a randomised, double-blind, placebo-controlled trial of adjunctive dexamethasone in Vietnamese adults and adolescents with TBM. Participants were recruited from two centres in Ho Chi Minh City, Vietnam: Pham Ngoc Thach Hospital and the Hospital for Tropical Diseases. Subjects were eligible to enter the trial if they were aged 15 years or older and had clinically suspected TBM. Details of the study design and analysis have been previously reported [1]. The primary outcome of the original study was death and disability at nine months after randomization. The protocol for this trial and the supporting CONSORT checklist are available as supporting information; see Protocol S1 and Checklist S1.

### Treatments

Adults previously untreated for tuberculosis received three months of daily oral isoniazid (5 mg/kg), rifampin (10 mg/kg), pyrazinamide (25 mg/kg; maximum dose 2g/day), and intramuscular streptomycin (20 mg/kg, maximum dose1g/day), followed by six months of isoniazid and rifampin at the same daily doses, according to Vietnamese national guidelines. Ethambutol (20 mg/kg, maximum dose 1.2 g /day) was substituted for streptomycin in HIV-infected patients, and was added to the regimen for the first three months in patients who had previously received treatment for tuberculosis. Antiretroviral therapy was not available at the time of the study.

Patients were randomized to receive treatment with either intravenous dexamethasone (0.3 to 0.4 mg/kg day) according to TBM grade at presentation and tapered over six to eight weeks, or identical placebo as previously described [1].

### Outcome at nine months

Treatment with dexamethasone was associated with a reduced risk of death in all patients (RR 0.69, 95% C.I. 0.52 to 0.92, p = 0.01). The relative risk for dying during the nine month follow up of the original study was 0.47 (95% C.I. 0.25 – 0.9 p = 0.02) in grade 1 TBM patients, 0.71 (95% C.I. 0.46 – 1.1 p = 0.11) in grade 2 TBM patients, and 0.81 (95% C.I. 0.51 – 1.29 p = 0.38) in grade 3 TBM patients. Dexamethasone was not associated with a significant reduction in the proportion of severely disabled patients [34 /187 patients (18.2%) among survivors in the dexamethasone group versus 22/159 patients (13.8%) in the placebo group, p = 0.27], or in the proportion of patients who had either died or were severely disabled after nine months (odds ratio 0.81, 95% C.I. 0.58 to 1.13, p = 0.22).

### Follow-up study

Participants were contacted by letter and/or telephone and invited to attend for a clinical assessment five years after randomization into the original study. If the patient had died prior to the follow-up assessment, the date and cause of death was requested from the relatives. Patients were assessed using a standard questionnaire and neurological examination. Neurological disability was assessed using standardized questionnaires ('two simple questions' and modified Rankin scale), as previously described [1]. Four survivors who were unable to attend the hospital gave verbal consent and were assessed by telephone interview. The primary outcome was overall survival during the five years of follow-up. The secondary outcome measures were disability status five years after randomization, and tuberculosis relapse (defined as re-treatment for tuberculosis) during the follow-up period. All data were recorded prospectively into individual case record forms entered into an electronic database (Microsoft Access 2003) and double-checked by the principal investigator prior to analysis.

### Ethics statement

Written informed consent was obtained for participation in the study. The study was conducted according to the ethical principles expressed in the Declaration of Helsinki. The study was approved by the institutional review boards of the participating hospitals (Scientific and Ethical Committee of the Hospital for Tropical Diseases, Scientific and Ethical Committee of Pham Ngoc Thach Hospital), and the Oxford Tropical Research Ethics Committee. The study was registered with *ClinicalTrials.gov* registry, identifier number NCT01317654.

### Statistical analysis

Survival curves were visualized using Kaplan-Meier estimators. We also displayed hazard rate estimates (number of events divided by person-years of follow-up) in both arms during the following time intervals: 0 to three, three to six, six to nine, nine to twelve months, and at yearly intervals thereafter. As supplementary graphical displays, we show the Nelson-Aaalen estimator of the cumulative hazard and smoothed hazard rate estimates based on a local bandwidth selection algorithm.

Proportional hazards were tested based on weighted Schoenfeld residuals. As there was strong evidence of non-proportional hazards for the treatment effect over time, we compared survival probabilities at fixed time points (yearly intervals from one to five years of follow-up) in all patients and by TBM grade, respectively. Kaplan-Meier estimates of survival probabilities were used and comparisons were based on Greenwood's variance formula. We also tested whether there was an interaction between TBM grade and dexamethasone treatment using a logistic regression, with outcome death prior to the respective time point, and excluding patients lost to follow-up. In addition, we fitted separate Cox survival models for the first three months of randomized treatment and the time thereafter. The following potential predictors of mortality were included in the Cox regression analysis: treatment group, admission TBM grade, HIV status, age, gender, haematocrit and serum sodium at baseline. The three-month cut-off was chosen because at this time point the initiation phase of tuberculosis treatment (and treatment with dexamethasone) had been completed. Of note, estimates (of hazards and hazard ratios) for the time period from month three onwards include only patients who survived beyond month three and this may select different subpopulations in the two treatment arms. Thus, causal interpretation of results from the latter time period should be applied with caution. All reported confidence intervals are two-sided 95% confidence intervals and analyses were performed with the statistical software R version 2.8.0 (R Foundation for Statistical Computing, Vienna, Austria) [16] and the companion R package muhaz version 1.2.5 (for smooth hazard estimation).

## Results

545 patients were randomized (274 in the dexamethasone arm, 271 in the placebo arm) between 4 April 2001 and 29 March 2003. 10/545 (2%) patients were lost to follow-up during the original nine-month follow-up period (5 in the dexamethasone group and 5 in the placebo group). A further 40 patients (18 in the dexamethasone group and 22 in the placebo group) were lost between nine months and five years, giving a total loss-to-follow-up of 9.2%. The median (IQR) timing of the follow-up visit for the survivors was 5.2 (4.7–5.5) years after randomization.

The baseline characteristics of the study participants are reported in the original publication [1]. In brief, the median age was 35 years (range 15 to 88 years), 61% were male, 18% were HIV-infected, and the distribution of patients with TBM grade 1, 2 and 3 was 32%, 45%, and 23%, respectively.

**Figure 1:**
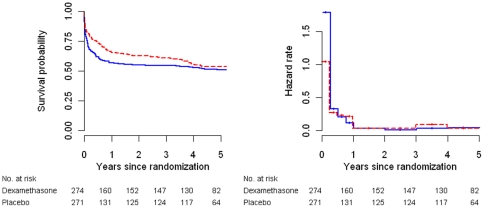
Kaplan-Meier curves (left panel) and hazard rate estimates (right panel) according to treatment group. The blue solid lines correspond to the placebo group, the dashed red lines to the dexamethasone group.

**Figure 2:**
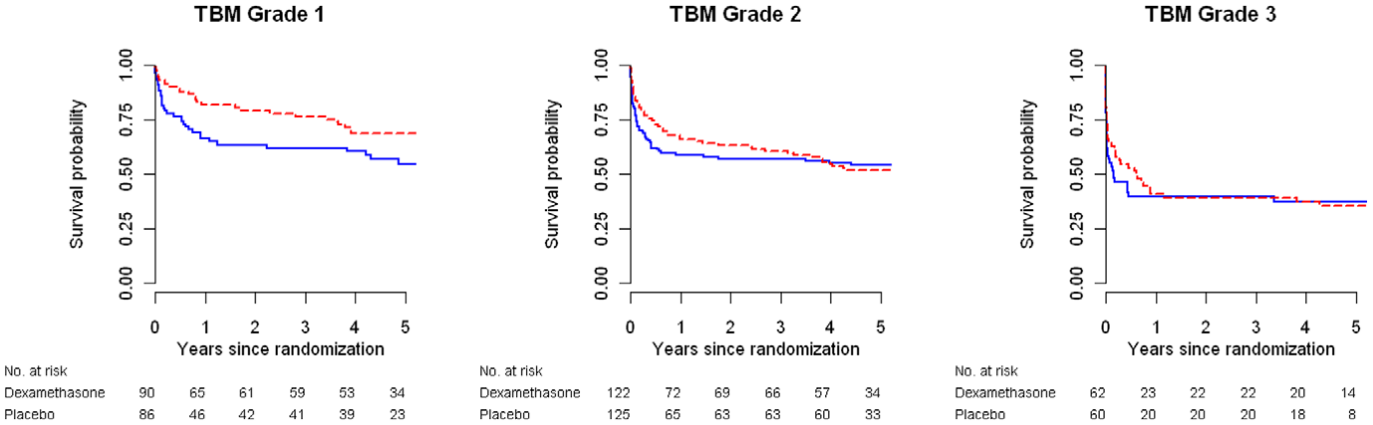
Kaplan-Meier curves by TBM grade. The blue solid lines correspond to the placebo group, the dashed red lines to the dexamethasone group.

A total of 249 subjects (121 in the dexamethasone arm and 128 in the placebo arm) died during the five-year follow-up period. 148 patients (59 dexamethasone group versus 89 placebo group) died in the first three months after randomization; 51 subjects (28 dexamethasone group versus 23 placebo group) died during months three to nine, and 50 patients (34 dexamethasone group versus 16 placebo group) died after month nine. Of the 50 subjects who died after nine months, 4 had a cause of death reported by the relatives (TBM, pneumonia and diabetes, myocardial infarction and asthma) and 46 had an unknown cause of death. There is no national mortality register in Vietnam and we were therefore unable to independently verify cause of death. Kaplan-Meier estimates of mortality in all patients are displayed in Figure 1 (left panel) and by TBM grade in Figure 2. Two-year survival probabilities tended to be higher in the dexamethasone arm (0.63 vs. 0.55; p = 0.07) but five-year estimates were similar (0.54 vs. 0.51, p = 0.51) in both groups (table 1). In grade 1 TBM, but not in grade 2 or grade 3 TBM the benefit of dexamethasone treatment tended to persist over time (five-year survival probabilities 0.69 vs. 0.55, p = 0.07) but there was no conclusive evidence of treatment effect heterogeneity by TBM grade (p = 0.36) (Table 1).

**Table 1:** Kaplan-Meier survival estimates at one to five years of follow-up for all patients and in the subgroups defined by TBM grade.

	Dexamethasone groupsurvival rate (95% CI)	Placebo groupsurvival rate (95% CI)	Difference (95% CI), p-value	p-value forheterogeneity*
**At 1 year**All patients (n = 545)	0.65 (0.60 to 0.71)	0.57 (0.51 to 0.63)	0.09 (0.00 to 0.17); p = 0.04	0.29
TBM grade 1 (n = 176)	0.82 (0.74 to 0.90)	0.66 (0.57 to 0.77)	0.15 (0.02 to 0.29); p = 0.02	
TBM grade 2 (n = 247)	0.66 (0.58 to 0.75)	0.59 (0.51 to 0.68)	0.07 (-0.05 to 0.19); p = 0.25	
TBM grade 3 (n = 122)	0.41 (0.30 to 0.55)	0.39 (0.29 to 0.54)	0.01 (-0.16 to 0.19); p = 0.88	
**At 2 years**All patients (n = 545)	0.63 (0.57 to 0.69)	0.55 (0.49 to 0.61)	0.08 ( 0.00 to 0.16); p = 0.07	0.25
TBM grade 1 (n = 176)	0.79 (0.71 to 0.88)	0.63 (0.54 to 0.75)	0.16 ( 0.02 to 0.29); p = 0.02	
TBM grade 2 (n = 247)	0.63 (0.55 to 0.72)	0.57 (0.49 to 0.66)	0.06 (-0.06 to 0.19); p = 0.33	
TBM grade 3 (n = 122)	0.39 (0.28 to 0.54)	0.39 (0.29 to 0.54)	0.00 (-0.18 to 0.17); p = 0.96	
**At 3 years**All patients (n = 545)	0.61 (0.55 to 0.67)	0.55 (0.49 to 0.61)	0.06 (-0.02 to 0.15); p = 0.15	0.26
TBM grade 1 (n = 176)	0.77 (0.68 to 0.86)	0.62 (0.52 to 0.74)	0.15 (0.01 to 0.29); p = 0.04	
TBM grade 2 (n = 247)	0.60 (0.52 to 0.70)	0.57 (0.49 to 0.66)	0.03 (-0.09 to 0.16); p = 0.59	
TBM grade 3 (n = 122)	0.39 (0.28 to 0.54)	0.39 (0.29 to 0.54)	0.00 (-0.18 to 0.17); p = 0.96	
**At 4 years**All patients (n = 545)	0.55 (0.50 to 0.62)	0.53 (0.47 to 0.59)	0.03 (-0.06 to 0.11); p = 0.56	0.35
TBM grade 1 (n = 176)	0.69 (0.59 to 0.80)	0.60 (0.50 to 0.72)	0.08 (-0.06 to 0.23); p = 0.27	
TBM grade 2 (n = 247)	0.55 (0.46 to 0.65)	0.55 (0.47 to 0.65)	0.00 (-0.13 to 0.12); p = 0.96	
TBM grade 3 (n = 122)	0.37 (0.27 to 0.52)	0.38 (0.27 to 0.52)	0.00 (-0.18 to 0.17); p = 0.98	
**At 5 years**All patients (n = 545)	0.54 (0.48 to 0.60)	0.51 (0.45 to 0.57)	0.03 (-0.06 to 0.12); p = 0.51	0.36
TBM grade 1 (n = 176)	0.69 (0.59 to 0.80)	0.55 (0.44 to 0.68)	0.14 (-0.01 to 0.29); p = 0.07	
TBM grade 2 (n = 247)	0.52 (0.43 to 0.62)	0.54 (0.46 to 0.64)	-0.02 (-0.15 to 0.11); p = 0.73	
TBM grade 3 (n = 122)	0.35 (0.25 to 0.50)	0.38 (0.27 to 0.52)	-0.02 (-0.20 to 0.15); p = 0.81	

* Interaction test of dexamethasone treatment with TBM grade based on a logistic regression for the outcome death and excluding censored patients

Survival curves corresponding to the two treatment arms got closer or crossed over with increasing time, a typical indication of non-proportional hazards. Indeed there was strong evidence of non-proportional hazards of the treatment effect over time both in an unadjusted analysis (p = 0.004) and in an analysis adjusted for TBM grade and HIV status (p = 0.007). Figure 1 (right panel) displays hazard rate estimates in different time intervals and displays a strong reduction in hazard due to dexamethasone during the first three months, but similar hazards in both arms from month three onwards. We also performed Nelson-Aalen estimate of cumulative hazards and hazard estimates based on kernel-based methods according to treatment arm (Figure S1).

Adjusted Cox regression analyses for both time periods are displayed in Table 2. For the first three months, the hazard of death was highly significantly reduced for subjects in the dexamethasone arm [hazard ratio (HR) = 0.62, 95% CI 0.44–0.88, p = 0.01]; TBM grade 2 or 3 and HIV infection were independent risk factors for death. In contrast, the hazard for subjects in the dexamethasone arm (and still alive by month 3) was increased after three months (HR = 1.50, 95% CI 1.00–2.26, p = 0.05); HIV infection, increasing age, and lower haematocrit, but not TBM grade, were independent risk factors for death. Antiretroviral therapy was not available during the time of the initial study (2001–2004) and there was limited availability thereafter. To our knowledge none of the HIV-infected patients received antiretroviral therapy.

**Table 2:** Cox regression analysis of mortality in months 0 to 3, and from month 3 onwards.</emph>

Covariate	Months 0 to 3			After month 3		
	HR	95% CI	p-value	HR	95% CI	p-value
Treatment with dexamethasone	0.62	0.44-0.88	0.01	1.50	1.00-2.26	0.05
Grade 2 TBM versus grade 1 TBM^£^	1.81	1.12-2.91	0.02	1.18	0.76-1.84	0.45
Grade 3 TBM versus grade TBM^£^	3.81	2.32-6.24	<0.001	1.44	0.81-2.57	0.22
HIV positive versus HIV negative^$^	2.15	1.39-3.32	<0.001	9.93	6.03-16.35	<0.001
HIV status unknown* vs HIV negative^$^	16.20	7.67-34.23	<0.001	-	-	-
Age (by +10 years)	1.06	0.95-1.17	0.29	1.28	1.13-1.45	<0.001
Haematocrit (by +10%)	0.95	0.73-1.23	0.68	0.68	0.49-0.94	0.02

*All patients with unknown HIV status died within the first 3 months.

£ Tests for interaction of TBM grade with treatment: p = 0.46 (months 0-3) and p = 0.18 (after month 3).

$ Tests for interaction of HIV status with treatment: p = 0.29 (months 0-3) and p = 0.17 (after month 3).

Gender and serum sodium were not significant in either model and didn't affect the other parameters. They were therefore omitted from the final mode.

As described in the methods section, our choice of the cut-point at 3 months for our Cox regression analysis was based on clinical grounds and the resulting Cox regression models for the two periods showed no evidence of non-proportional hazards for the study drug effect (p = 0.66 for the first period, p = 0.18 for the second period). However, exploratory data-driven analyses suggest a somewhat higher cut-point of around 7 months with values up to 9 months for the true change point being compatible with the data. Importantly however, the selection of the cut-point had little impact on covariate effects. For example, a cut-point selection of 9 months lead to the same significant covariates for both periods except that baseline hematocrit was no longer significant for the period after 9 months (p = 0.28) but became borderline significant for the early period (p = 0.08).

Disability status at the five-year follow-up was available for 493 (90.5%) of patients and is displayed in table 3. There was no significant association between randomized treatment and disability status at five years (p-value linear trend test = 0.32). There was, however, a highly significant association between disability status at nine months and five years (p<0.001). Indeed the proportions of patients with severe disability or death at five years were 1.2% and 11.3%, respectively, in 168 patients with good outcome at nine months, compared with 12.5% and 20% in 80 patients with intermediate outcome at nine months, and 50.0% and 32.6% in 46 patients with severe disability at nine months.

**Table 3:** Disability status at 9 months and 5 years post-randomisation.

	Dexamethasone group	Placebo group
**Outcome at 9 months**	**N = 274**	**N = 271**
Good	104 (38%)	95 (35.1%)
Intermediate	49 (17.9%)	42 (15.5%)
Severe	34 (12.4%)	22 (8.1%)
Dead	87 (31.8%)	112 (41.3%)
**Outcome at 5 years**	**N = 250**	**N = 243**
Good	69 (27.6%)	61 (25.1%)
Intermediate	43 (17.2%)	36 (14.8%)
Severe	17 (6.8%)	18 (7.4%)
Dead	121 (48.4%)	128 (52.7%)

Of 246 individuals who were alive at the time of the five-year interview, only three (all in the placebo group) reported that they had received re-treatment for TB. It was not possible to assess whether patients who had died between nine months and five years had been re-treated for tuberculosis prior to death.

## Discussion

Our original trial [1] showed that adjunctive dexamethasone treatment improved survival in patients with TBM at nine months. The present study shows a survival benefit of dexamethasone treatment for up to two years of follow-up. The benefits on long-term (five-year) survival are inevitably less clear, and may be confined to patients with grade I TBM, and dexamethasone was not associated with improvement in disability status at nine months or five years post-randomisation.

We managed to obtain follow-up data from the vast majority (91.2%) of patients at five years. Although the five-year survival estimates were similar in the two arms, there did appear to be a persistent benefit of dexamethasone in the patients with grade 1 TBM. One possible explanation is that dexamethasone may prevent the development of neurological complications in this group of patients, whereas it has limited effect on those who present with established neurological deficits (grade 2 or 3 TBM). This has important clinical implications and re-emphasises the need to diagnose and treat TBM promptly, before neurological complications ensue. Although TBM may have an indolent presentation, once a patient comes to the attention of medical services, the condition should be managed as a medical emergency, as clinical outcome is critically dependent on the TBM grade at the initiation of therapy.

We observed that the Kaplan-Meier survival curve for all patients converged over the five-year follow-up period, suggesting non-proportional hazards. This was confirmed by a Cox survival hazards model which indicated that dexamethasone had a highly significant benefit during the first three months of follow-up which was partially offset by a higher subsequent mortality amongst three-month survivors in the dexamethasone group. A possible explanation is that dexamethasone saves lives acutely, but leaves some individuals with severe neurological sequaelae, which subsequently lead to death. Two baseline variables, HIV status and increased TBM grade, were found to be associated with death during the initial 3 months of treatment. When we examined the follow-up period beyond three months, HIV status, low haematocrit and increased age were found to be independently associated with death. 

Disability status at nine months and five years was assessed using the modified Rankin score and the 'two simple questions'. These tools are commonly used for assessment of disability in stroke patients and give an indication of the extent to which patients require physical assistance with activities of daily living, a somewhat crude measure of disability. Examination of the disability data at nine months reveals that severe disability was more frequent in the dexamethasone arm compared with the placebo arm (12.4% versus 8.1%), and that intermediate outcome showed a similar pattern (17.9% versus 15.5%, respectively). There was no difference in the number of patients with TBM grade 1 who had severe disability at nine months (four in each treatment arm); this low rate of disability at nine months may explain why the survival benefit in grade 1 TBM appears to persist out to five years. For patients with grade 2 and 3 TBM there were more severely disabled patients at 9 months in the dexamethasone arm, compared with the placebo arm: 19 versus 11 (grade 2 TBM) and 11 versus 7 (grade 3 TBM). By five years intermediate outcome remained more frequent in the dexamethasone arm compared with the placebo (17.2% versus 14.8%) but the rates of severe outcome were similar (6.8% versus 7.4%). Thus dexamethasone may be preventing death at nine months but leaving more patients disabled; these disabled patients go on to die over the course of the follow-up period.

We acknowledge several limitations of our study. The primary outcome measure was all-cause mortality at five years and it was not possible to determine whether the cause of death was related to TBM or not. Secondly, we did not formally assess neuro-cognitive function in the patients, which may have been able to detect more subtle differences in disability. Thirdly, our Kaplan-Meier curves and analyses suggest a larger benefit of dexamethasone treatment in TBM grade 1 but tests for heterogeneity of the treatment effect in different TBM grades were not significant. While such tests have low power, we cannot conclusively rule out that the observed heterogeneity is due to chance alone. The sample size in the original study was powered to detect a difference in the primary endpoint outcome at nine months; no sample size was calculated in relation to the five-year follow up outlined in this study. Finally, there are no specialist neuro-rehabilitation services available in Vietnam and this may influence long-term outcome of disabled patients; these results may therefore not be comparable to outcome for disabled TBM survivors in settings where such facilities exist.

The findings of our original study indicated that dexamethasone improved survival at nine months in all patients with TBM, although it had no effect on neurological disability. This study demonstrated a benefit of dexamethasone up to two years of follow-up but could not demonstrate a five year survival benefit, which may be confined to patients with less severe disease.

## Supporting Information

Figure S1 Cumulative hazard (left panel) and smoothed hazard rate estimates (right panel) according to treatment group. The blue solid lines correspond to the placebo group, the dashed red lines to the dexamethasone group. The blue dash-dotted and the red dotted lines display the cumulative hazards for the two groups in TBM grade 1 patients only.
(TIF)

Protocol S1 Trial Protocol.
(DOC)

Checklist S1 CONSORT Checklist
(DOC)
