# Supplementary figures and images for: Dexamethasone and Long-Term Outcome of Tuberculous Meningitis in Vietnamese Adults and Adolescents
Source: PLoS One. 2011 Dec 8;6(12):e27821. doi: 10.1371/journal.pone.0027821 (PMC3234244; doi:10.1371/journal.pone.0027821)

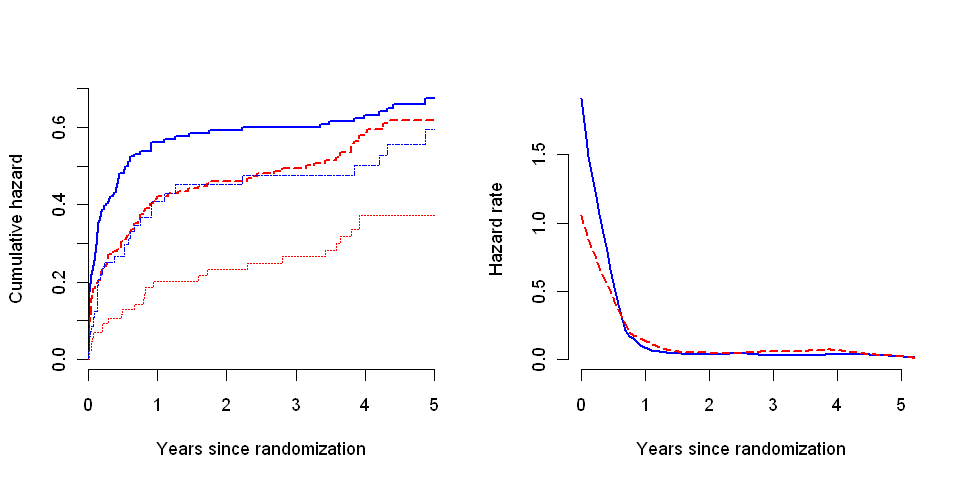

Supplement: Figure S1 — Cumulative hazard (left panel) and smoothed hazard rate estimates (right panel) according to treatment group. The blue solid lines correspond to the placebo group, the dashed red lines to the dexamethasone group. The blue dash-dotted and the red dotted lines display the cumulative hazards for the two groups in TBM grade 1 patients only. (TIF) [file pone.0027821.s001.tif]
