# Supplementary material for: Dexamethasone and Long-Term Outcome of Tuberculous Meningitis in Vietnamese Adults and Adolescents
Source: PLoS One. 2011 Dec 8;6(12):e27821. doi: 10.1371/journal.pone.0027821 (PMC3234244; doi:10.1371/journal.pone.0027821)
Supplement: Protocol S1 — Trial Protocol. (DOC) [file pone.0027821.s002.doc]

# PROTOCOL

# Study Of The Long Term Outcome Of Tuberculous Meningitis In Vietnamese Adults Treated With Adjunctive Dexamethasone (OXTREC 017-06)

# Investigators

# Dr Estee Torok (principal investigator, OUCRU)

Dr Tran Thi Hong Chau (HTD)

Dr Nguyen Duc Bang (PNT)

Dr Nguyen Thi Bich Yen (PNT)

Dr Hoang Thi Quy (PNT)

Dr Tran Tinh Hien (HTD)

Professor Jeremy Farrar (OUCRU)

# Introduction

Tuberculous meningitis is the severest form of infection with *Mycobacterium tuberculosis,*

causing death or severe neurologic deficits in more than half of those affected in spite of antituberculosis chemotherapy [1, 2]. Early studies suggested that corticosteroids reduced cerebrospinal fluid inflammation and time to recovery in patients with tuberculous meningitis, but the studies were too small to confirm any effect on survival [3-7]. Concern remained that corticosteroids might reduce the case fatality rate but increase the number of disabled patients [8]. Randomized trials performed in Egypt [9] and South Africa [10] provided evidence that corticosteroids improved survival in children with severe disease and probably reduced neurologic sequelae. A meta-analysis of all randomized controlled trials of corticosteroids for tuberculous meningitis suggested that corticosteroids were effective in reducing the risk of death in children but not in patients over 14 years of age although only six trials involving a total of 595 patients (158 adults) met the inclusion criteria, and there were no data on human immunodeficiency virus (HIV) co-infected patients [11]. The authors concluded that small numbers of patients, poor concealment of the treatment-group assignments, and publication bias could account for the positive results, and that studies in patients with HIV infection and studies of a size large enough to assess morbidity and the case fatality rate were required. We therefore conducted a randomized, double-blind, placebo-controlled trial to determine whether adjunctive dexamethasone therapy reduced the risk of death or disability in Vietnamese adults (≥15 years old).

The results of the study were as follows. A total of 545 patients were randomly assigned to groups that received either dexamethasone (274 patients) or placebo (271 patients). Only 10 patients (1.8 percent) had been lost to follow-up at nine months of treatment. Treatment with dexamethasone was associated with a reduced risk of death (relative risk, 0.69; 95 percent confidence interval, 0.52 to 0.92; P=0.01). It was not associated with a significant reduction in the

proportion of severely disabled patients (34 of 187 patients [18.2 percent] among survivors in the dexamethasone group vs. 22 of 159 patients [13.8 percent] in the placebo group, P=0.27) or in the proportion of patients who had either died or were severely disabled after nine months (odds ratio, 0.81; 95 percent confidence interval, 0.58 to 1.13; P=0.22). The treatment effect was consistent across subgroups that were defined by disease-severity grade (stratified relative risk of death, 0.68; 95 percent confidence interval, 0.52 to 0.91; P=0.007) and by HIV status (stratified relative risk of death, 0.78; 95 percent confidence interval, 0.59 to 1.04; P=0.08). Significantly fewer serious adverse events occurred in the dexamethasone group than in the placebo group (26 of 274 patients vs. 45 of 271 patients, P=0.02). We therefore concluded that adjunctive treatment with dexamethasone improved survival in Vietnamese patients over 14 years of age with tuberculous meningitis but probably did not prevent severe disability[12].

One of the limitations of previous studies of patients with tuberculous meningitis is the duration of follow-up after the end of tuberculosis treatment. There are scanty data available about relapse rates and no information about long term outcome. Although the dexamethasone study showed a reduction in mortality we do not know if this benefit persists and, if so, for how long. We also do not know the outcome of patients who were severely disabled at the end of treatment. We therefore propose to conduct a simple study to determine the long-term outcome in patients recruited to the dexamethasone study 5 years after recruitment into the original study.

The questions that we seek to answer are:

1. Does the difference in mortality between the 2 groups persist at 5 years?
2. Are there differences in neurological disability between the 2 groups at 5 years?
3. Are there differences in TB relapse rates between the 2 groups?

## Methods

The study will be conducted at the Hospital for Tropical Diseases (HTD) and Pham Ngoc Thach Hospital (PNT) in Ho Chi Minh City, Vietnam

All patients who were alive at the end of the dexamethasone study (n=340) will be eligible to participate in this long-term follow-up study.

Patients will be sent an invitation letter (Appendix 1) containing a simple questionnaire to complete and return. Those that do not return the questionnaire will be contacted by telephone or traced through the local health services.

Written informed consent (Appendix 2) will be obtained from all patients or their relatives (if the patient is unable to give consent).

Those patients who agree to participate and are able to travel to the hospital will be offered an appointment and their transport costs will be reimbursed.

Those patients who agree to participate in the study, but who are unable to travel to hospital to attend an appointment, will be assessed at home by a doctor.

The assessment will consist of a simple questionnaire, a clinical examination and a blood test.

Data will be collected in individual case record forms and entered into a computer database.

The study will be co-ordinated by Dr Estee Torok (Oxford University Clinical Research Unit), Dr Tran Thi Hong Chau (Hospital for Tropical Diseases) and Dr Nguyen Duc Bang (Pham Ngoc Thach Hospital)

## Outcome measures

The outcome measures for the study are:

1. Survival at 5 years
2. Neurological disability at 5 years
3. TB relapse rate

## Statistical analysis

This will be performed by Kasia Stepniewska using STATA. The following analyses will be performed:

1. Kaplan-Meier survival estimates and log rank test
2. Comparison of disability scores between 2 groups by chi square test
3. Comparison of TB relapse rate between 2 groups by chi square test

## Funding

The study will be funded by the Wellcome Trust through the Oxford University Clinical Research Unit Grant.

The total budget for the study is USD 10, 826.25 which will be divided between the 2 hospitals according to the number of patients followed up.

The breakdown for the estimated maximum costs of the study is as follows:

1. Stationary costs (letter paper, envelopes, stamps) = USD 50
2. Telephone costs: 2 phone cards @ $20 per month x 24 months = USD 480
3. Patient travel costs
   1. HCMC urban = 150,000 VND x 150 = 22,500,000 VND = USD 1406.25
   2. HCMC suburban  = 200,000 VND x 100 = 20,000,000 VND = USD 1250
   3. Provinces  = 400,000 VND x 50 = 20,000,000 VND = USD 1250
4. Cost of home visit (if required) = 300,000 VND x 40 = 12,000,000 VND = USD 750
5. Cost for completing CRF: $5 per patient x 340 = USD 1700 (if all alive)
6. Venesection: $2 per patient x 340 = USD 340
7. Cost for 3 supervisors at PNT (Dr Bang, Dr Yen, Dr Quy) = 3 x $50 per month x 24 months = USD 3,600

**References**

1. Girgis, N.I., et al., *Tuberculosis meningitis, Abbassia Fever Hospital-Naval Medical Research Unit No. 3-Cairo, Egypt, from 1976 to 1996.* Am J Trop Med Hyg, 1998. **58**(1): p. 28-34.

2. Hosoglu, S., et al., *Predictors of outcome in patients with tuberculous meningitis.* Int J Tuberc Lung Dis, 2002. **6**(1): p. 64-70.

3. Shane, S.J. and C. Riley, *Tuberculous meningitis: combined therapy with cortisone and antimicrobial agents.* N Engl J Med, 1953. **249**(21): p. 829-34.

4. Ashby, M. and H. Grant, *Tuberculous meningitis treated with cortisone.* Lancet, 1955. **268**(6854): p. 65-6.

5. Lepper, M.H. and H.W. Spies, *The present status of the treatment of tuberculosis of the central nervous system.* Ann N Y Acad Sci, 1963. **106**: p. 106-23.

6. O'Toole, R.D., et al., *Dexamethasone in tuberculous meningitis. Relationship of cerebrospinal fluid effects to therapeutic efficacy.* Ann Intern Med, 1969. **70**(1): p. 39-48.

7. Escobar, J.A., et al., *Mortality from tuberculous meningitis reduced by steroid therapy.* Pediatrics, 1975. **56**(6): p. 1050-5.

8. Parsons, M., *The treatment of tuberculous meningitis.* Tubercle, 1989. **70**(2): p. 79-82.

9. Girgis, N.I., et al., *Dexamethasone adjunctive treatment for tuberculous meningitis.* Pediatr Infect Dis J, 1991. **10**(3): p. 179-83.

10. Schoeman, J.F., et al., *Effect of corticosteroids on intracranial pressure, computed tomographic findings, and clinical outcome in young children with tuberculous meningitis.* Pediatrics, 1997. **99**(2): p. 226-31.

11. Prasad, K., J. Volmink, and G.R. Menon, *Steroids for treating tuberculous meningitis.* Cochrane Database Syst Rev, 2000(3): p. CD002244.

12. Thwaites, G.E., et al., *Dexamethasone for the treatment of tuberculous meningitis in adolescents and adults.* N Engl J Med, 2004. **351**(17): p. 1741-51.
